# Anemia and Its Treatment

**Published:** 1897-04

**Authors:** L. A. Felder

**Affiliations:** Atlanta, Ga.


### ANEMIA AND ITS TREATMENT.

By L. A. FELDER, M.D.,

ATLANTA, GA.

Within the past few months I have had under my care and treatment several cases of anemia that may prove interesting to the readers of THE ATLANTA MEDICAL AND SURGICAL JOURNAL. The pathological anatomy, various causes and symptoms of anemia have been so thoroughly sifted and presented to the medical profession by able writers, that I will not attempt to add to the able articles that have been written by more experienced hands, after much hard labor and abundant experience. We, as practitioners, are made to feel quite often, the need of more light on the subject. The various conditions that exist and are to be combated in anemia are truly a study to the general practitioner, and should be of equal importance to the specialists also. Especially so in anemia does this apply to the dermatologist, for it is quite often the case that he has to combat with anemic conditions in many cases of diseases of the skin. First, in the treatment of anemia, as in all diseases that the human being falls a victim to, we by the exclusion of everything else but the real cause can with safety make an honest effort to remove the same. After the cause has been clearly defined and treatment for its eradication well founded and established, we next look for remedial agents that will restore to the deficient, diseased tissues and organs of the body the loss they have sustained from disease.

In anemia and anemic conditions, the skin, in a great many cases, suffers particularly in comparison with other organs of the body. To supply different tissues and organs of the body with remedial agents that exist in health and are deficient from disease is by no means an easy task.

In two cases of anemia that I have recently treated and will report, the skin in both cases had to have special attention. In one case there was development of boils on different parts of the body, confined largely to axilla and back of neck. The boils were clearly due to anemic conditions and poor capillary blood supply to parts. Not enough manganese existed in the skin and hair follicles forced there by a good healthy circulation of rich blood to keep the skin in healthy condition; hence the formation of boils. In the second case there were liver-colored splotches on back of hands, with still feebler capillary circulation, and this was the more marked case of anemia. At first in both these cases I had to combat conditions such as loss of appetite, and inability of both patients to assimilate proper amount of nourishment. After dieting patients on proper nutritious foods, and usual course of stomach tonics with some improvements in general health, the diseased condition of the skin, in both cases, still existed. For relief of this condition I prescribed manganese with iron and peptones as prepared by Gude, dessertspoonful after each meal in wineglass of milk, with results in both cases most gratifying. Iron manganese with peptones in this preparation I have found in all cases of anemia and anemic conditions where iron and manganese are indicated, a most excellent preparation. And in no formulæ have I ever prescribed two drugs that are more readily assimilated and taken up into the system than in this preparation.

Mr. B., age 45; spare build; leads a sedentary life; previous health record up to last fall fairly good. November, 1896, had severe case of la grippe, complicated with acute bronchitis; slow recovery; loss of appetite; food poorly digested; passed particles of undigested food for three weeks prior to seeing me; had been taking tonics and cod-liver oil. Some improvement in general health. Digestion still bad; bowels irregular. Close examination, patient extremely weak and anemic; quick weak pulse, 90; flabby tongue; liver-colored splotches on back of hands; skin white and prominent blue veins; conjunctiva white, and capillary circulation poor; every evidence of marked anemia. I at once commenced patient on dessertspoonful of Gude's pepto-manganese in wineglass of sweet milk after each meal. Liver inactive, for relief of which I prescribed half hour before each meal small doses of phosphate of sodium to increase secretion of bile; proper nutritious diet with very little starchy foods; small amount of stimulants taken all during treatment; at end of three weeks good healthy circulation, disappearance of liver-colored splotches on back of hands and sound recovery.

Mr. B., age thirty-five; medium build, good health record up to six months ago; condition very simlar to case above mentioned in every particular, except in liver-colored splotches on skin; formation of boils as result of feeble blood supply to skin. Treatment in this case same, and at end of second week splendid results and sound recovery.

---

A SCIENTIST has discovered that persons who get up early get mad sooner than those who do not. It does not need a scientist to tell us that. A man who is awakened at 6 o'clock and ordered to get up and build the fire has a right to get mad. His wife has certainly not anything to get mad about.—*Puck.*

